# Natural Alkaloid Compounds as Inhibitors for Alpha-Synuclein Seeded Fibril Formation and Toxicity

**DOI:** 10.3390/molecules26123736

**Published:** 2021-06-19

**Authors:** Simona S. Ghanem, Hend S. Fayed, Qi Zhu, Jia-Hong Lu, Nishant N. Vaikath, Janarthanan Ponraj, Said Mansour, Omar M. A. El-Agnaf

**Affiliations:** 1 Neurological Disorders Research Center, Qatar Biomedical Research Institute (QBRI), Hamad Bin Khalifa University (HBKU), Qatar Foundation, Doha 34110, Qatar; sghanem@hbku.edu.qa (S.S.G.); hfayed@hbku.edu.qa (H.S.F.); nvaikath@hbku.edu.qa (N.N.V.); 2 Division of Biological and Biomedical Sciences (BBS), College of Health & Life Sciences (CHLS), Hamad Bin Khalifa University (HBKU), Doha 34110, Qatar; 3 State Key Laboratory of Quality Research in Chinese Medicine, Institute of Chinese Medical Sciences, University of Macau, Macau 999078, China; imzhuqi@163.com (Q.Z.); jiahonglu@um.edu.mo (J.-H.L.); 4 Qatar Environment and Energy Research Institute (QEERI), Hamad Bin Khalifa University (HBKU), Doha 34110, Qatar; jponraj@hbku.edu.qa (J.P.); smansour@hbku.edu.qa (S.M.)

**Keywords:** α-synuclein, Parkinson’s disease, seeded fibril formation

## Abstract

The accumulation and aggregation of α-synuclein (α-syn) is the main pathologic event in Parkinson’s disease (PD), dementia with Lewy bodies, and multiple system atrophy. α-Syn-seeded fibril formation and its induced toxicity occupy a major role in PD pathogenesis. Thus, assessing compounds that inhibit this seeding process is considered a key towards the therapeutics of synucleinopathies. Using biophysical and biochemical techniques and seeding-dependent cell viability assays, we screened a total of nine natural compounds of alkaloid origin extracted from Chinese medicinal herbs. Of these compounds, synephrine, trigonelline, cytisine, harmine, koumine, peimisine, and hupehenine exhibited in vitro inhibition of α-syn-seeded fibril formation. Furthermore, using cell viability assays, six of these compounds inhibited α-syn-seeding-dependent toxicity. These six potent inhibitors of amyloid fibril formation and toxicity caused by the seeding process represent a promising therapeutic strategy for the treatment of PD and other synucleinopathies.

## 1. Introduction

Parkinson’s disease (PD) is a neurodegenerative disorder that affects the substantia nigra region of the brain, resulting in a massive loss of dopaminergic neurons [1,2,3,4,5]. Neuropathologically, PD is associated with a buildup of cytoplasmic inclusions known as Lewy bodies (LB) and Lewy neurites (LN) [6,7], with the main constituent being alpha synuclein (α-syn) protein. α-Syn intracellular inclusions are also a distinctive feature of other neurodegenerative diseases, including multiple system atrophy (MSA) and dementia with Lewy bodies (DLB) [8]. Under physiological conditions, this small (14 kDa) acidic protein exists unfolded with no or little ordered secondary structure [9]. Upon different environmental stimuli and genetic factors, native and soluble α-syn can form oligomers and eventually develop into well-ordered stable fibrils [10]. The aggregation of α-syn studied in vitro is a nucleation-dependent process, described by an initial lag phase, followed by an elongation (growth phase) to reach the steady-state phase [11,12,13]. The lag phase is characterized by an accumulation of soluble species lacking fibrillar conformation, known as oligomers, which develop into mature amyloid fibrils with time [14,15,16,17]. The formation of these pathogenic β-sheet structures contributes to the toxicity and neurodegeneration leading to the onset and progression of the disease.

The most challenging aspect in PD is the absence of tools to halt the continuous progression of neurodegeneration. PD is commonly managed through alleviating the symptoms by increasing dopaminergic nerve activity including dopamine agonists or suppressing dopamine metabolism [18]. These options only offer symptomatic relief and do not prevent the continuous loss of dopaminergic neurons in the substantia nigra. In accordance with the rise of PD incidence due to aging population and the importance of α-syn aggregation in PD pathogenesis, diverse studies have invested in a wide range of strategies to target α-syn aggregation and its associated cytotoxicity [19,20]. Some of these strategies are decreasing α-syn protein expression or improving clearance mechanisms [21] and passive or active immunization [20,22,23]. Moreover, peptides have gained much attention in the field of neurodegenerative diseases due to their potential to impede the protein–protein interactions and inhibit fibril formation [24,25,26,27,28,29] or block the seeding process of amyloid formation [30].

Alkaloids are naturally occurring organic compounds primarily found in plants. These natural products have a wide spectrum of pharmacological activities including antioxidant, antimicrobial, anti-tumor, anti-inflammatory, hypoglycemic, and neuroprotective properties [31,32,33,34,35,36,37,38]. Interestingly, alkaloids exhibit neuroprotective characteristics with a wide mode of action to lessen the development of many neurodegenerative diseases in the preclinical setting, such as PD, Alzheimer’s disease (AD), Huntington disease (HD), epilepsy, dementia and memory impairment, and psychological disorders, making them valuable therapeutic agents for neurological disorders [39,40,41,42]. Despite the few challenges and limitations of natural products, such as their low bioavailability and rapid metabolism, there are reports of overcoming these concerns by the use of nanotechnology and nanocarrier-based approaches [43,44,45,46]. Consistent with the fact that these natural compounds provide a better, cheaper, and safer alternative with minimal side effects compared to synthetic compounds, there is a vital need to search for new natural products.

Taking all this together, and given the ability of α-syn aggregates in cell-to-cell transmission by seeding aggregation of endogenous α-syn in unaffected neurons [47], the aim of the present study was to screen nine herbal alkaloid medicinal compounds for their ability to inhibit α-syn-seeded fibril growth and its related α-syn-induced toxicity. They include synephrine, trigonelline, cytisine, harmine, koumine, hupehenine, peimisine, corydaline, and rotundine. We conclude that six out of the nine compounds have the ability to inhibit α-syn seeded polymerization and limit its induced toxicity, thus demonstrating the great potential of effective herbal therapeutic options for PD and other synucleinopathies.

## 2. Results

### 2.1. The Effect of Alkaloid Compounds on In Vitro Induced α-Syn Seeded Fibril Formation

**Figure 1:**
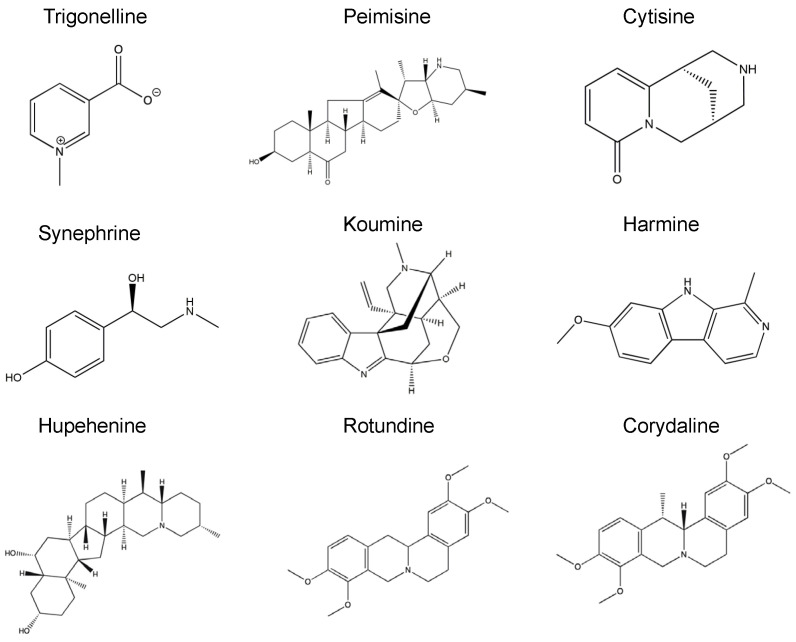
Chemical structures of nine natural alkaloid compounds to test their effect on α-syn seeded fibril formation and toxicity.

**Figure 2:**
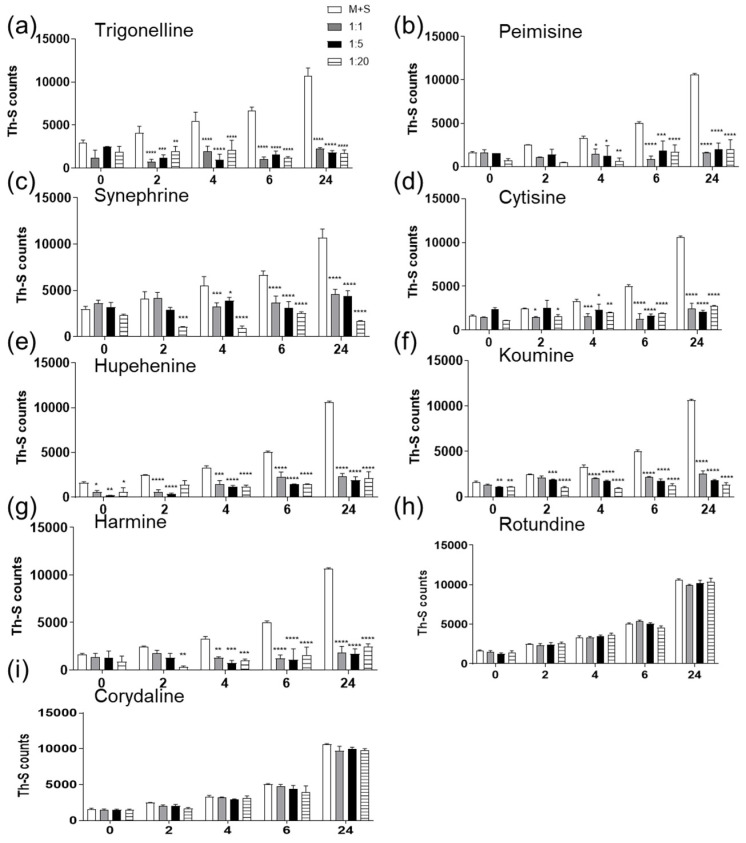
Effect of alkaloid compounds on α-syn seeded aggregation (**a**–**i**). Fibril formation analysis for each sample was performed using Th-S assay. α-Syn monomers solution (25 µM) were incubated alone or with tested compound at different molar ratios (1:1, 1:5, 1:20) for 24 h at 37 °C with continuous shaking at 800 rpm. Means ± standard deviations are shown. Statistical analysis was performed using Two-way ANOVA with Tukey’s multiple comparison testing (**** *p* < 0.0001, *** *p* < 0.001, ** *p* < 0.01; * *p* < 0.05).

In recent years, naturally derived alkaloids have been involved in the management of neurodegenerative diseases and have shown high potential in inhibiting amyloid aggregation [36,39,48,49]. Given the importance of seeds in the pathological amyloid formation in vitro and in vivo [2,50,51,52,53], we screened nine Chinese herbal alkaloid compounds for their effect on seeded induced α-syn aggregation. All of the tested compounds are natural and of herbal origin, with their chemical structures illustrated in Figure 1. To study any possible inhibitory effect on α-syn fibril growth, 25 µM of α-syn was incubated for 24 h at 37 °C with α-syn seeds alone (1 µM) or α-syn seeds previously incubated for 1 h with the tested compound at molar ratios of seeds: compounds 1:1, 1:5, and 1:20. Fibril formation was measured by Thioflavin S assay (Th-S). The results of seven compounds showed a clear decrease in the percentage of relative seeded aggregation to the monomers and seeds group, demonstrating a reduction in α-syn fibrils (Figure 2a–g). The seven compounds are synephrine, trigonelline, cytisine, harmine, koumine, hupehenine, and peimisine. Although it showed an inhibitory effect on fibril formation, synephrine showed a milder inhibition compared to the remaining compounds. On the contrary, corydaline and rotundine did not present any effect on α-syn aggregation (Figure 2h,i). Moreover, as illustrated in Supplementary Figure S1, fibril formation analysis by Th-S assay of each compound alone was tested immediately, showing no variation in Th-S counts between all groups, excluding the possibility of quenching.

**Figure 3:**
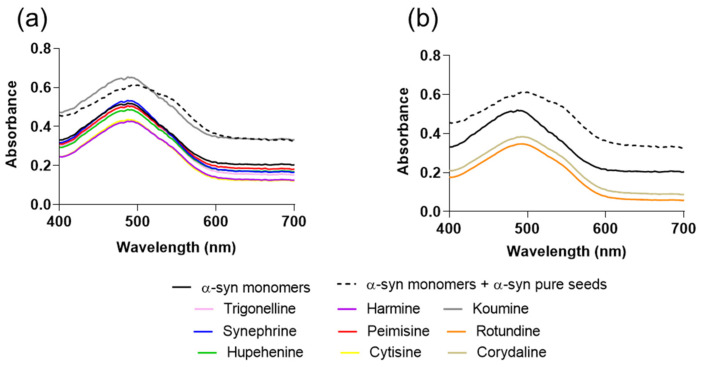
Congo red (CR) absorbance spectra in the presence of alkaloid compounds (**a**,**b**). Means ± standard deviations are from triplicates of one experiment.

To further analyze α-syn fibril formation, we performed Congo Red (CR) binding assay on these samples. Congo red is a dye that has high affinity to α-syn fibrils. The presence of amyloid fibrils can be detected by monitoring the shift of the dye absorbance maximum from 490 nm to 540 nm. The samples of α-syn incubated in the presence of synephrine, trigonelline, cytisine, harmine, koumine, hupehenine, and peimisine for 24 h and at molar ratio 1:20 showed no CR absorption shift, with similar absorption wavelengths to the group of monomeric α-syn alone (Figure 3a). This illustrates that these compounds inhibit the formation of structures with β-sheet conformation. On the other hand, the CR absorption maximum of monomers and seeds group exhibited a pronounced shift, with comparable results to the groups incubated with corydaline and rotundine (Figure 3b). Once again, this suggests the inability of these two compounds to inhibit amyloid fibril growth. The obtained CR maxima wavelength for each group is listed in Supplementary Table S1.

**Figure 4:**
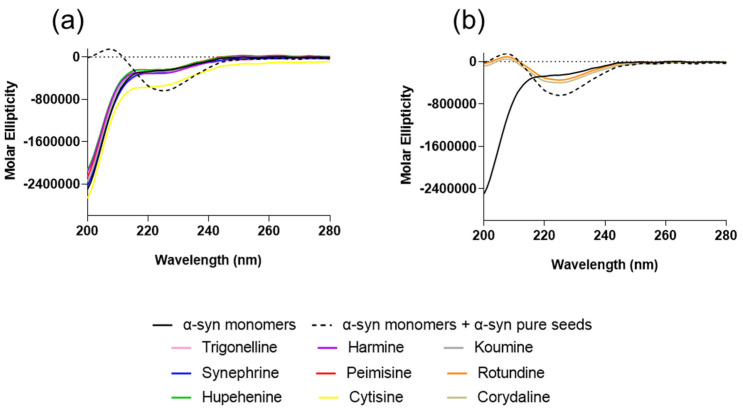
Circular dichroism (CD) spectra showing secondary structural changes in the presence of alkaloid compounds (**a**,**b**). Means ± standard deviations are from triplicates of one experiment.

In order to characterize the secondary structure and detect the presence of β-sheet structure of the aggregates, CD spectra were studied. The CD spectra were obtained from the prepared solutions after an incubation of 24 h at a 1:20 molar ratio of seeds: compound. The spectra of α-syn monomers and seeds incubated with the compounds synephrine, trigonelline, cytisine, harmine, koumine, hupehenine, and peimisine were flat at wavelengths from 210 to 250 nm, showing typical random coil profiles, similar to α-syn monomers alone (Figure 4a). This is consistent with the analyses of the Th-S aggregation assay and CR results that suggest the anti-fibrillogenic activity of these natural compounds. On the contrary, samples incubated with rotundine and corydaline showed comparable β-sheet spectra to the monomers and seeds group with a negative peak centered at 225 nm, indicating the presence of a β-sheet structure and their inability of inhibiting α-syn fibril formation (Figure 4b). The obtained CD maxima and minima wavelength for each group is listed in Supplementary Table S2. Additionally, CD spectra performed on each compound alone did not show a signal (Supplementary Figure S2).

**Figure 5:**
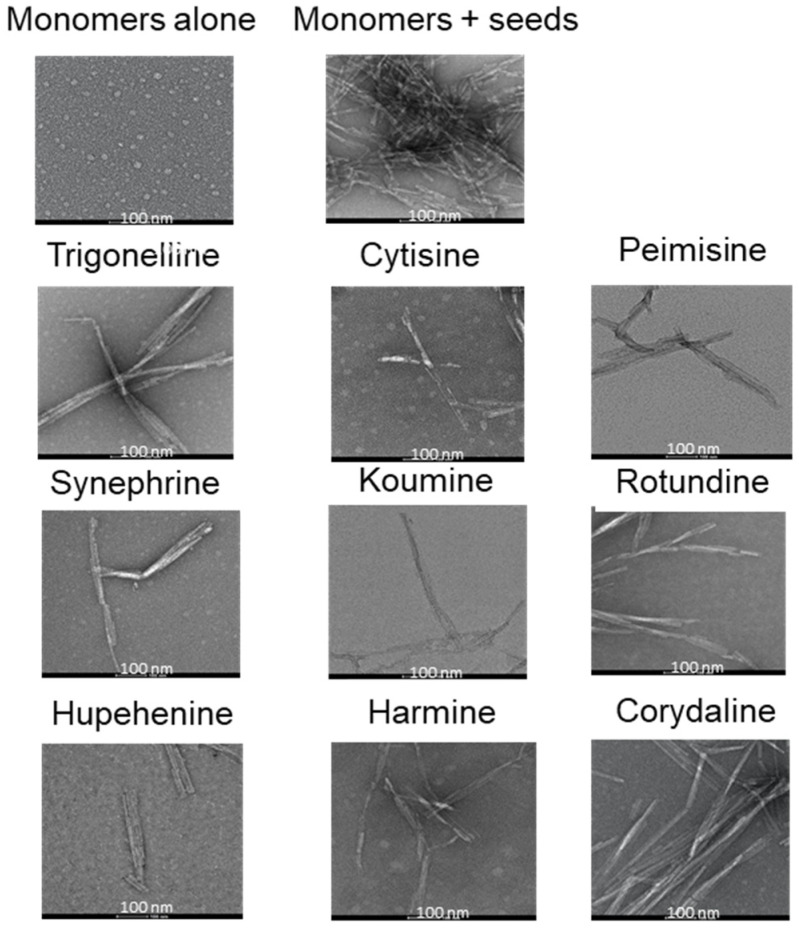
Electron microscopy images reflecting fibril formation of α-syn incubated with or without alkaloid compounds at molar ratio 1:5. Scale bar, 100 nm.

Additional confirmation of the compounds’ inhibitory effect on fibril formation was reflected by electron microscopy. TEM images were taken for all samples incubated with various compounds at a molar ratio of 1:5 for 24 h. The TEM images shown in Figure 5 are a representation of what is seen throughout the grid. α-Syn incubated with seeds alone showed dense meshes of long and thick fibrils. Upon treating with synephrine, trigonelline, cytisine, harmine, koumine, hupehenine, or peimisine, few thin bundled fibrils were scattered. On the other hand, incubation with rotundine and corydaline showed a higher density of fibrils forming mesh-like structures throughout the grid.

### 2.2. The Effect of Alkaloid Compounds on α-Syn Seeded Induced Toxicity

**Figure 6:**
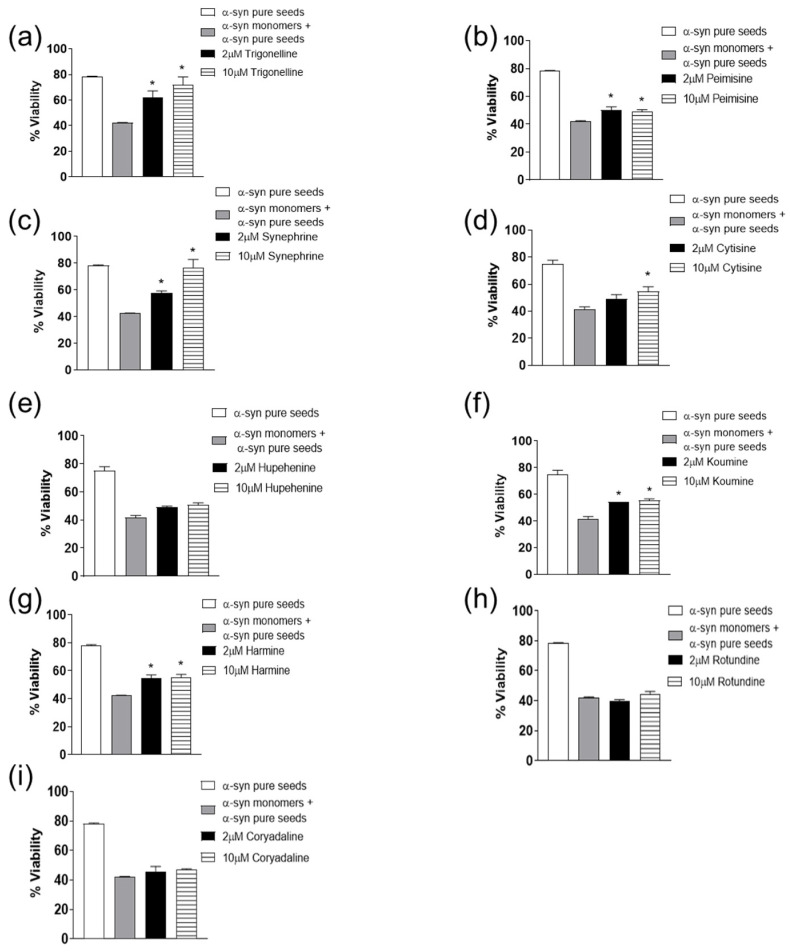
Estimation of viability on WT SH-SY5Y human neuroblastoma cells by the MTT assay. The results are expressed as percentage of the untreated cells. The cells were treated with 0.01 µM of α-syn pure seeds and 10 µM of α-syn monomers with or without 2 and 10 µM of compounds (**a**–**i**) for 48 h prior to MTT addition (average of 3 wells ± standard deviation). Statistical analysis was performed using One-way ANOVA with Tukey’s multiple comparison testing (* *p* < 0.05).

Inhibiting fibril growth and seeding capacity plays a central role in protecting against α-syn-induced toxicity [53,54,55]. The resultant toxicity should be taken into consideration when finding new inhibitors for α-syn fibril growth. Thus, MTT assay using WT SH-SY5Y neuroblastoma cells was employed for the assessment of the cytotoxic effect of α-syn species with or without the tested compounds [11,53]. As shown in Figure 6, and consistent with the previous data of synephrine, trigonelline, cytisine, harmine, koumine, and peimisine in inhibiting α-syn seeds induced aggregation (Figure 6a–g), these compounds also reduced the toxicity induced by α-syn seeds, leading to a significant increase in cell viability. On the other hand, hupehenine did not demonstrate a significant reduction in toxicity. Moreover, rotundine and corydaline showed no protection against α-syn-induced toxicity as shown in Figure 6h,i, with similar results to the monomers and seeds group, likely due to their inability to inhibit α-syn fibril formation. The cell toxicity effect of each alkaloid compound alone (1, 5, 10 µM) was also assessed (Supplementary Figure S3). All compounds showed minimal effect on cell viability except harmine, which demonstrated an approximate 50% cytotoxic effect.

## 3. Discussion

PD is a neurodegenerative disease with an incidence expected to rise tremendously in the upcoming years [56]. In accordance with many studies, the pathogenesis of PD and other related disorders is linked to α-syn misfolding and aggregation [1,16,57]. In its native state, α-syn is unfolded, soluble, and nontoxic; however, during misfolding it becomes insoluble, forming aggregates in several brain regions [58,59,60]. Given that the current treatment of PD is only moderate symptomatic relief, there is an urgent need to develop more effective treatments. Since seeded fibril formation and its related toxicity have been implicated in PD pathogenesis, many studies have focused on halting this process by developing and screening molecules for potential treatment strategies [61,62,63,64]. As previously shown, natural and alkaloid compounds have been considered potent amyloid aggregating inhibitors and neuroprotective agents [36,48,65,66]. Considering this, we screened nine alkaloid herbal Chinese compounds for their effect on α-syn seeding fibril formation and toxicity: synephrine, trigonelline, cytisine, harmine, koumine, hupehenine, peimisine, corydaline, and rotundine.

The results presented in this study suggest that synephrine, trigonelline, cytisine, harmine, koumine, hupehenine, and peimisine act as potent inhibitors of α-syn-seeded aggregation at all three molar ratios, as validated by Th-S assays. Electron microscopy studies showed the inhibitory effects of these alkaloids on α-syn, where only very few dense meshes of fibrils were evident, accounting for the minimal Th-S counts and anti-fibrillogenic activity detected by CR and CD analysis. These results are in accordance with previous reports that described trigonelline’s importance as a major alkaloid of fenugreek seeds that induces neurite outgrowth in vitro in human neuroblastoma SK-N-SH cells [67]. Moreover, it was stated that trigonelline also improves cognition and alleviates neuronal loss in Alzheimer’s disease rat models and has neuroprotective potential in PD rodent models [68,69]. Interestingly, cytisine has also shown positive effects in studies where it reduced MPTP-induced dopamine depletion in mouse models [34]. Additionally, harmine was previously reported as a potent compound that degrades α-syn [36]. These two latter compounds, cytisine and harmine, were also shown to cross the blood–brain barrier [70,71,72], suggesting important future implications. The remaining two compounds, rotundine and corydaline, exerted no inhibitory effect on fibril formation, as illustrated by the time-dependent increase in Th-S counts. This was also confirmed by the prominent absorption maximum shift of CR, the presence of β-sheet structure by CD, and the dense meshes of α-syn fibrils by electron microscopy.

Fibrils with high β-sheet content and their binding to cell membranes cause membrane permeabilization and a change in calcium homeostasis, ultimately causing cytotoxicity [11,73,74]. In regard to these compounds, only synephrine, trigonelline, cytisine, harmine, koumine, and peimisine reduced the neurotoxic effect provoked by the seeding process, demonstrating an increase in the cell viability, possibly due to the inhibition of the amyloid aggregation. Although these compounds showed a neuroprotective effect in WT SH-SY5Y neuroblastoma cells, rotundine and corydaline did not curb the seeded induced neurotoxicity. Despite the anti-fibrillogenic activity of hupehenine, it did not restrain the seeded induced toxicity, possibly due to its inefficiency in WT SH-SY5Y neuroblastoma cell model. Previous research has also reported similar results highlighting the effect of natural compounds on decreasing fibril growth and cytotoxicity [75,76].

In conclusion, the promising data in this study reflect the neuroprotective and anti-fibrillation effects of six potent herbal compounds that can be considered candidates for PD therapeutics, after thoroughly understanding their inhibitory effect and studying them on PD animal models. This represents a major advancement towards potential therapeutic approaches for PD and other related disorders.

## 4. Materials and Methods

### 4.1. Preparation of Recombinant α-Syn Protein

Full-length recombinant human α-syn was expressed in Escherichia coli BL21 (DE3) using the bacterial expression vector pRK172, as previously reported [77]. Following expression and sedimentation, the bacterial pellets from 1 L of Terrific broth (TB) were homogenized and sonicated in 50 mL of high-salt buffer (0.75 M NaCl, 10 mM Tris, pH 7.6, 1 mM EDTA) containing a cocktail of protease inhibitors (Thermo Scientific, Waltham, MA, USA), heated to 100 °C for 10 min, and centrifuged at 5300× *g* for 20 min. The solution was dialyzed overnight against the buffer used for gel filtration chromatography (50 mM NaCl, 10 mM Tris, pH 7.6, 1 mM EDTA), following which the volume was reduced to 5 mL using a Pierce protein concentrator (10 K MWCO; ThermoFisher Scientific, Waltham, MA, USA) according to the manufacturer’s instructions. All proteins were purified by size exclusion using a Superdex 200 gel filtration column (GE Healthcare, Little Chalfont, UK). The clean fractions were pooled, exchanged with a buffer (10 mM Tris pH 7.6, 25 mM NaCl, 1 mM EDTA, 1 mM PMSF) for ion exchange chromatography by dialysis overnight, and applied onto a HiTrap Q column (GE Healthcare) and eluted in 10 mM Tris pH 7.6 using a linear gradient of 0.025–1.0 M NaCl. For preparation of α-syn monomers, the protein went through 100 KDa filters to remove any high molecular weight proteins. Purified fractions were pooled, and protein concentrations were determined using the Pierce BCA protein assay kit (ThermoFisher Scientific) [77]. To obtain α-syn monomers, the protein solution was passed through 100 KDa filters to remove high-molecular-weight proteins. Monomeric α-syn (100 µM) was aggregated at 37 °C for 7 days with continuous shaking at 800 rpm. Pure fibrils were prepared upon spinning the crude α-syn fibril sample at 14,000 rpm for 15 min at 4 °C using a microfuge (Eppendorf). After that, supernatant was discarded, and the pellet was washed to be resuspended in 1xPBS. For pure seed preparation, the pure fibrils were fragmented on ice by ultrasonication using a Sonic Ruptor 250. For protein quantification, samples were denatured with an equal volume of 6 M Guanidine-HCl and quantified using Pierce BCA protein assay kit (ThermoFisher Scientific).

### 4.2. Alkaloid Compounds

Synephrine, trigonelline, cytisine, harmine, koumine, hupehenine, peimisine, corydaline, and rotundine were the nine compounds tested in this study. They are all natural alkaloids and above 98% pure. These compounds were kindly provided from Chengdu Must Bio-Technology Co. and Shanghai Yuanye Bio-Technology Co. from Chengdu and Shanghai, China. The compounds were all dissolved in 100% dimethyl sulfoxide (DMSO). Sample solutions were prepared such that the final concentration of DMSO in each sample solution was not more than 1% [61].

### 4.3. Thioflavin-S (Th-S) Assay

In a total volume of 100 µL, α-syn pure seeds (1 µM) were incubated either in PBS alone or in combination with the compounds at different molar ratios (seed: compound), 1:1, 1:5, and 1:20 for 1 h at 37 °C with continuous shaking at 300 rpm. α-Syn monomers (25 µM) were added to the reaction mixture at a final volume of 500 µL and incubated at 37 °C with continuous shaking at 800 rpm. Samples (10 µL) at different time points were diluted in 40 µL of Th-S (20 µM) in PBS, and the mixture was dispensed in a 384-well, untreated black microplate (Nunc). Fluorescence was measured in a microplate reader (Perkin Elmer Envision, Waltham, MA, USA) with the excitation and emission wavelengths at 450 and 510 nm, respectively.

### 4.4. Congo Red (CR) Binding Assay

Congo red (Sigma-Aldrich, Saint Louis, MO, USA) (20 μM) was prepared in PBS (pH 7.4) and filtered through a 0.45 μm filter. Samples of α-syn (5 μM), aged alone or with compounds at 1:20 molar ratio after a 24 h incubation, were mixed with Congo red (final concentration 5 μM). The UV absorbance spectrum was recorded between 400 and 700 nm in a spectrophotometer (DU-800, Beckman-Coulter, Indianapolis, IN, USA) using 10-mm quartz cuvettes (Hellma Analytics-Germany, Mullheim, Germany). Congo red alone was used as blank.

### 4.5. Circular Dichroism (CD)

An amount of 5 µM of α-syn monomers and seeds with or without compounds at 1:20 molar ratio were used for CD measurement after a 24 h incubation. The far-UV CD spectra (between 250 and 190 nm) were recorded on a Chirascan™ CD Spectrometer (Applied Photophysics, Leatherhead, UK) with a step size of 0.1 nm using a cuvette with of path length of 1 mm. The scan for each sample was repeated 5 times, and all spectra were corrected by subtracting the background value of the buffer.

### 4.6. Transmission Electron Microscopy (TEM)

Samples (5 µL) from the aggregation assay were added on Formvar-coated 400-mesh copper grids (Agar Scientific, Essex, UK), followed by brief fixation with 0.5% glutaraldehyde and washing with double distilled water. Finally, samples were negatively stained with 2% uranyl acetate (Sigma) and imaged with an FEI Talos F200C TEM electron microscope at 200 keV. TEM was performed on samples incubated with the compounds for 24 h and at a molar ratio, seeds: compound 1:5.

### 4.7. Tissue Culture of WT SH-SY5Y

Human dopaminergic neuroblastoma cells (WT SH-SY5Y) (ATCC) were cultured in Dulbecco’s MEM/Nutrient Mix F-12 (1:1) (HyClone) containing 15% FBS (HyClone), 1% penicillin-streptomycin (P/S; 10,000 U/mL penicillin, 10 mg/mL streptomycin-Sigma), and supplemented with 1% non-essential MEM amino acid supplement (Gibco) and 2 mM freshly prepared glutamine. The cells were maintained at 37 °C in a humidified incubator with 5% CO_2_/95% air.

### 4.8. Measurement of Cell Viability

WT SH-SY5Y cells were suspended in culturing medium and plated at a density of 15,000 cells (100 µL /well) in a 96-well plate. After 24 h, the medium was replaced with 100 μL of MEM-RS (HyClone) serum-free medium containing the different solutions of α-syn species (pure seeds and monomers) with or without the compounds, diluted in serum-free media to obtain the desired concentrations. The cells were then allowed to grow for 48 h. A total of 20 μL of 3-(4, 5-dimethylthiazol-2-yl)-2,5-diphenyltetrazolium bromide (MTT) (Sigma-Aldrich, USA) (6 mg/mL) in PBS was dispensed into each well, and the plate was incubated at 37 °C for 4.5 h. The MTT-containing medium was carefully removed and replaced with 100 μL/well of lysis buffer (15% SDS, 50% *N*,*N*-dimethylformamide, pH 4.7). The lysis buffer was incubated at 37 °C overnight. The absorbance values at 590 nm were measured in a microplate reader (Perkin Elmer).

### 4.9. Statistical Analysis

The data are presented as mean ± standard deviation. Statistical analysis was performed using One-way ANOVA, followed by Tukey’s multiple comparison test by the GraphPad Prism software (version 8.3.0).

## Data Availability

Data is contained within the article and supplementary material.

## Supplementary Materials

The following are available online, Figure S1: Effect of alkaloid compounds on α-syn aggregation, Figure S2: Circular dichroism (CD) spectra of alkaloid compounds alone (20 µM), Figure S3: Effect of alkaloid compounds on cell toxicity (A–I), Table S1: Maxima wavelength values (nm) of Congo red (CR) absorbance spectra for all groups, Table S2: Maxima and minima wavelength values (nm) of Circular dichroism (CD) spectra for all groups.
