# Supplementary material for: Natural Alkaloid Compounds as Inhibitors for Alpha-Synuclein Seeded Fibril Formation and Toxicity
Source: Molecules. 2021 Jun 19;26(12):3736. doi: 10.3390/molecules26123736 (PMC8234408; doi:10.3390/molecules26123736)
Supplement: Supplementary file 1 [file molecules-26-03736-s001.zip › molecules-1215670-supplementary.pdf]

Group	Maxima Wavelength (nm)
α-syn monomers	479
α-syn monomers + α-syn pure seeds	494
Cytisine	486
Harmine	485
Hupehenine	485
Koumine	486
Peimisine	486
Synephrine	486
Trigonelline	486
Corydaline	494
Rotundine	493

Supplementary Table 1: Maxima wavelength values (nm) of Congo red (CR) absorbance spectra for all groups.

Group	Maxima Wavelength (nm)	Minima Wavelength (nm)
α-syn monomers	274	200
α-syn monomers + α-syn pure seeds	208	226
Cytisine	279	200
Harmine	253	200
Hupehenine	253	200
Koumine	253	200
Peimisine	263	200
Synephrine	274	200
Trigonelline	267	200
Corydaline	208	225
Rotundine	208	223

Supplementary Table 2: Maxima and minima wavelength values (nm) of Circular dichroism (CD) spectra for all groups.

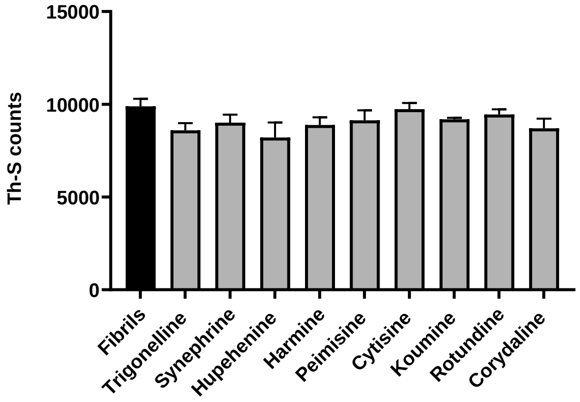

Supplementary Figure 1: Effect of alkaloid compounds on α-syn aggregation. Fibril formation analysis for each sample was performed using Th-S assay. α-Syn fibril-solution (5 µM) was incubated alone or with tested compound (20 µM), and tested immediately at time point 0. The assay was performed in triplicates, and the means ± standard deviations are shown.

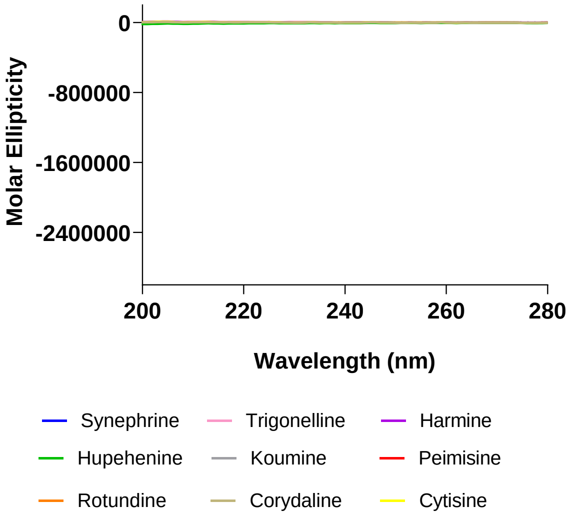

Supplementary Figure 2: Circular dichroism (CD) spectra of alkaloid compounds alone (20 µM). Means ± standard deviations are from triplicates of one experiment.

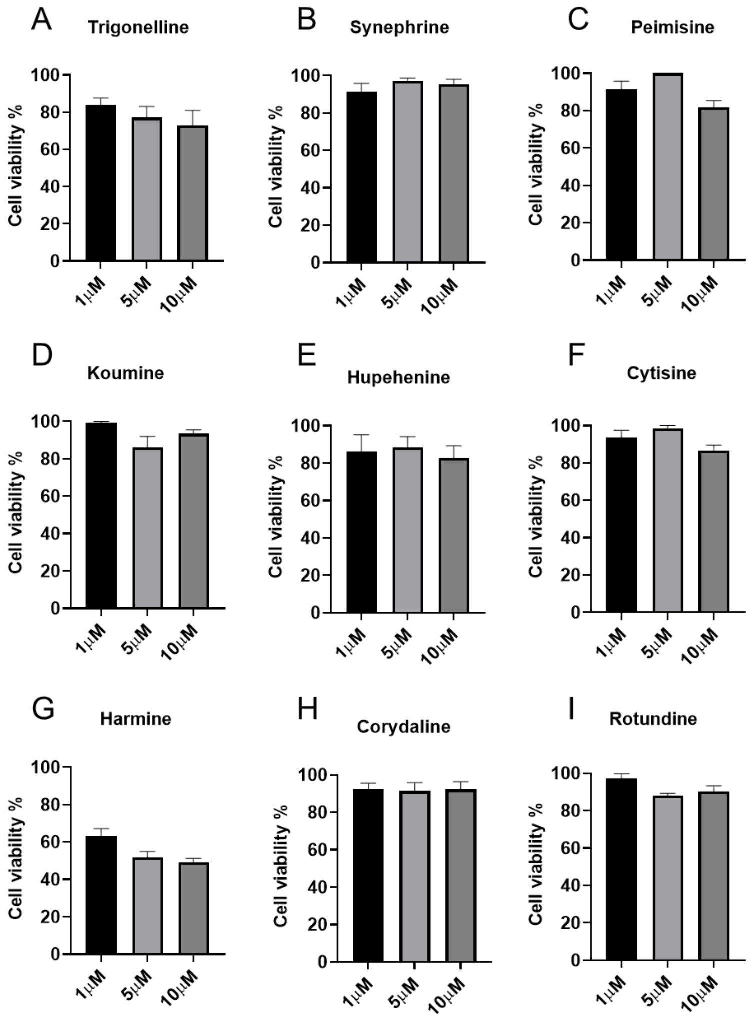

Supplementary Figure 3: Effect of alkaloid compounds on cell toxicity (A – I). The viability of WT SH-SY5Y human neuroblastoma cells was evaluated using MTT cell viability assay. Cells were incubated for 48 hours with compounds (A) Trigonelline, (B) Synephrine, (C) Peimisine, (D) Koumine, (E) Hupehenine, (F) Cytisine, (G) Harmine, (H) Corydaline and (I) Rotundine at different molar concentrations (1µM, 5µM and 10µM). The results are expressed as percentages of the average of the untreated cells. Values are expressed as means ± standard deviations.
